# Significant role of savolitinib in a case of advanced gastric cancer with abnormal mesenchymal‐epithelial transition factor (MET): A case report

**DOI:** 10.1097/MD.0000000000032072

**Published:** 2022-12-02

**Authors:** XinCheng He, GaiLi An

**Affiliations:** a Department of Internal Medicine Oncology, Shaanxi Provincial People’s Hospital, Shaanxi, China.

**Keywords:** advanced gastric cancer, MET gene, savolitinib

## Abstract

Gastric cancer is a common and lethal malignancy worldwide. It lacks specific clinical symptoms during the early stages, and when detected, the optimal surgical opportunity is lost. Chemotherapy alone offers limited benefits in advanced inoperable disease or postoperative recurrence. Gastric cancer is a heterogeneous tumor involving multiple gene regulations; thus, multi-target combination therapy is the trend in research. The c-MET protein is a tyrosine kinase receptor belonging to the MET family, encoded by the MET proto-oncogene. After binding with its ligand, the hepatocyte growth factor, MET activates cellular signaling pathways in proliferation, motility, migration, and invasion. In addition, it may be abnormally activated in cancers via mutation, amplification, and protein overexpression.

**Patient concerns and diagnosis:**

We report a 35-year-old male with advanced gastric cancer and bone metastasis who was intolerant to chemotherapy. He was in poor general condition, with thrombocytopenia and anemia.

**Interventions and outcome:**

Next-generation sequencing (NGS) suggested MET gene amplification in the tumor. After savolitinib treatment, the condition improved significantly without noticeable adverse reactions and maintained a progression-free status for 14 weeks.

**Lessons:**

This case report provides evidence for MET tyrosine kinase inhibitors in treating gastric cancer patients with MET gene amplification. It also shows that MET detection is a target in gastric cancer.

## 1. Introduction

Receptor tyrosine kinases (RTKs) and their downstream signaling pathways have attracted significant attention in the research for tumor target therapy. The mesenchymal‐epithelial transition (MET) factor protein is an RTK encoded by the proto-oncogene c-MET. The hepatocyte growth factor (HGF) binding secreted from stromal cells to MET influences biological processes such as cell proliferation, migration, and differentiation.^[1]^ In 2021, savolitinib was approved in China for treating patients who had progressed after platinum-containing chemotherapy and were intolerant to locally advanced or metastatic non-small-cell lung carcinoma (NSCLC) with a MET exon 14 skipping mutation (METex14m).^[2]^ In gastric cancer, the most common mechanism of aberrant activation of the MET pathway is high MET protein expression, and overexpression of MET protein suggests a poor prognosis. Xiao et al found that patients with positive c-MET expression had significantly shorter progression-free survival (PFS) and overall survival (OS) than those with negative expression.^[3]^ However, there is a lack of extensive reports on the efficacy of savolitinib in patients with MET-amplified advanced gastric cancer. We reported a case of advanced gastric cancer with bone metastasis. The next-generation sequencing (NGS) suggested MET amplification. The patient had an uncontrolled and rapidly-progressive disease after immunotherapy plus chemotherapy. Patients had performance status (PS) scores of 4 points and were intolerant to standard chemotherapy. The targeted therapy with savolitinib for 14 weeks showed that the PS score improved (by 1 point). Imaging suggested improved disease with no significant adverse effects, indicating a better quality of life and improved overall condition and prognosis. The positive outcome of this case provides a new treatment option for gastric cancer patients with MET amplification.

## 2. Case report

A 35-year-old male was presented to the hospital with “choking, abdominal pain, and distension” after a meal. He had no significant past medical history. However, he had a family history of cancer. His maternal grand-uncle died of lung cancer, the grandmother died of breast cancer, one maternal aunt died of lung cancer, and another maternal aunt developed colon cancer.

**Figure 1.**
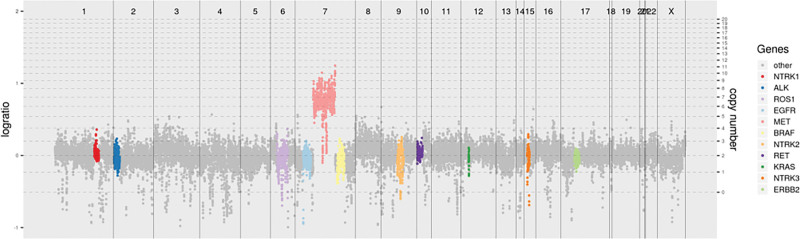
NGS results show MET amplification. MET = mesenchymal-epithelial transition, NGS = next-generation sequencing.

In October 2021, a gastroscopy and biopsy suggested a poorly differentiated adenocarcinoma of the gastric corpus, stage cT4N+Mx. On December 16, 2021, after 2-cycles of neoadjuvant therapy with camrelizumab plus SOX (oxaliplatin plus tegafur), the patient underwent radical surgery with total gastrectomy and adhesiolysis. Postoperative histopathology revealed ulcerative, poorly differentiated adenocarcinoma with signet ring cells mucinous carcinoma, stage pT4aN3bM1. In addition, the human epidermal growth factor receptor 2 (HER2) was negative, ki67 was 40%, MSS, Epstein‐Barr virus-encoded small RNA (EBER) negative, programmed cell death ligand 1 (PD-L1) tumor proportion score (TPS) < 1%, combined positive score (CPS) < 1%, low tumor mutational burden (TMB-L), with MET amplification (7) (Fig. 1).

The patient subsequently received five cycles of SOX combined with nivolumab. The platelet (PLT) count decreased to 20 × 10^9^/L during the treatment.

**Figure 2.**
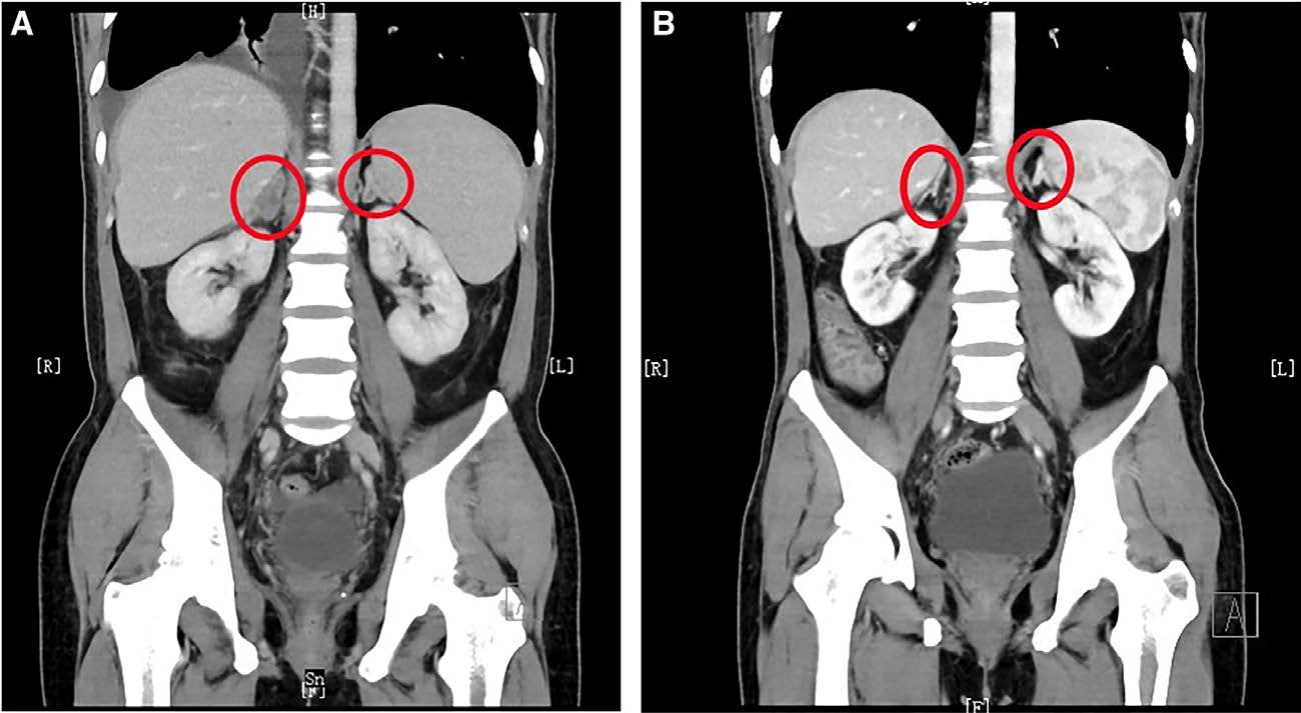
Before treatment, CT showed that bilateral adrenal glands were significantly thickened and decreased in density, and low-density shadows were seen after enhanced scanning (A). After treatment, CT showed that the left adrenal gland was thickened, the enhancement was not uniform after enhanced scanning, and the abnormal enhancement shadow of the original right adrenal gland disappeared (B). Peritoneal and pelvic effusions were reduced.

In May 2022, lumbosacral pain appeared and gradually increased after six months of surgery and chemotherapies. He received five cycles of lumbosacral radiotherapy and one cycle of concurrent denosumab. Oxycodone hydrochloride 70 mg/day and topical fentanyl transdermal patch 4.2 mg/72h were provided for pain management. On June 15, 2022, the hemoglobin (Hb) was 3.0 g/dL and PLT 11 × 10^9^/L. A blood transfusion was administered. The lumbosacral pain aggravated, and the oxycodone hydrochloride was increased to 70 mg/12 h. On July 7, 2022, the red blood cell (RBC) count was 2.34 × 10^12^/L, Hb 6.6 g/dL, PLT 42 × 10^9^/L, carcinoembryonic antigen (CEA) 73.6 ng/mL, cancer antigen 72-4 (CA72-4) > 250.00 IU/mL, and CA19-9 362 was U/mL. The CT revealed bilateral adrenal metastasis, the uneven density of lumbar-2 vertebral bodies, and abdominal and pelvic ascites (Fig. 2A). Physical examination showed a weight of 50.0 kg (BMI 16.3 kg/m^2^), anemic face, pale conjunctivae, no obvious bleeding spots, and ecchymosis. The patient’s general condition was poor (PS 4 points), with intermittent fever of up to 38.5 ℃. The lumbosacral pain became worse. Bone marrow biopsy revealed no metastatic tumor cells. Repeated transfusion of RBCs, platelets, and TPO was administered to regulate the anemia and thrombocytopenia. The lumbosacral pain worsened, and he developed a pulmonary infection.

On July 6, 2022, after nine months of surgery and chemo-radiotherapies, considering earlier intolerance to chemotherapy and MET amplification in the tumor, oral savolitinib 400 mg/day was started, together with supportive treatment for symptomatic relief. After seven days of savolitinib treatment, the lumbosacral pain was gradually relieved, pain medications were discontinued, anemia improved, and fever subsided. The general condition improved significantly (PS score was 1–2 points). The RBC was 3.3 × 10^12^/L, Hb 9.0 g/dL, and PLT 221 × 10^9^/L.

After 39 days of savolitinib treatment, RBC was 3.85 × 10^12^/L, Hb 11.3 g/dL and PLT 137 × 10^9^/L, carcinoembryonic antigen (CEA) 1.1 ng/mL, cancer antigen 72-4 (CA72-4) 41.4 IU/mL, and CA19-9 18.5 U/mL. The CT showed that the left adrenal nodule had reduced, the right adrenal nodule disappeared, the density of the lumbar-2 vertebral bodies was uneven, and the abdominal and pelvic effusion was less than before (Fig. 2B). The patient’s general condition improved significantly (PS score was 1 point), with no fever, and lumbosacral pain had subsided. He had gained 56.50 kg (BMI 20.0 kg/m^2^), and there was no pallor, bleeding points, or ecchymosis.

**Figure 3.**
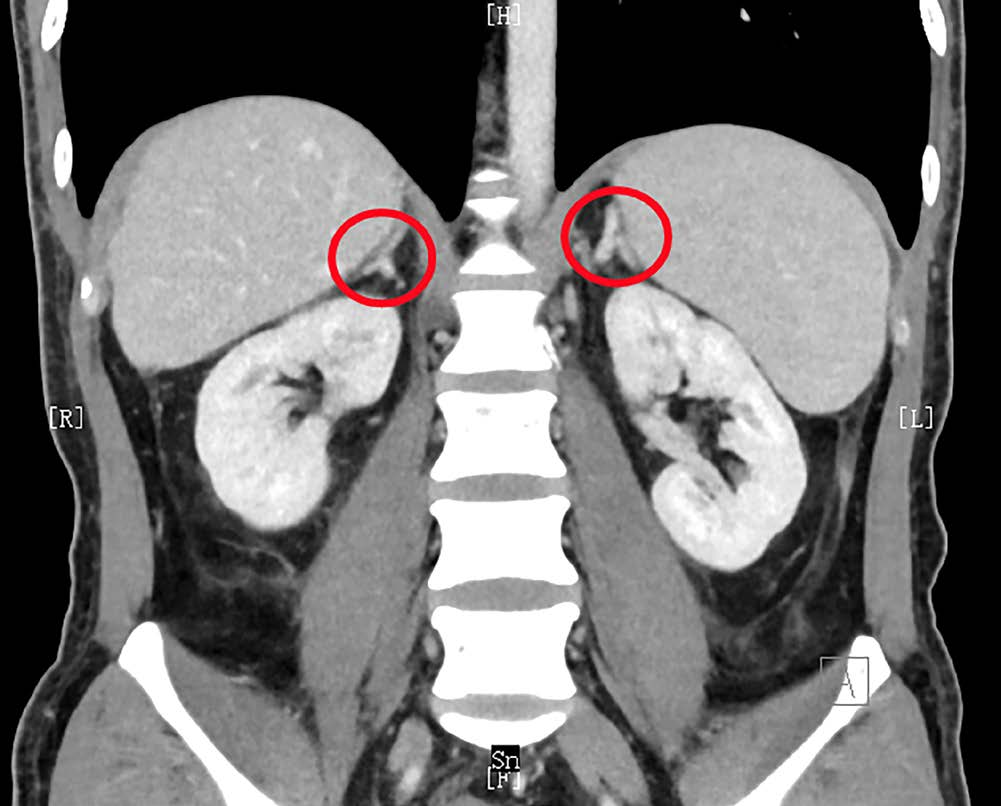
CT reexamination after 14 weeks of treatment showed no progress.

According to Response Evaluation Criteria in Solid Tumors (RECIST) version 1.1, the patient achieved partial response (PR), that is, a decrease in size of ≥10% or a decrease in tumor density (HU) ≥ 15% on CT, no new lesions, no obvious progression of the non-measurable disease. After 14 weeks of savolitinib treatment, during treatment, the patient survived and CT reexamination showed that the disease did not progress (Fig. 3).

## 3. Discussion

This case report of a 35-year-old male with MET-amplified advanced gastric cancer and metastasis who was intolerant and did not respond to conventional chemotherapy showed a significant antitumor effect with improvement in general condition, better PS scores, relief of lumbosacral pain, and regression of bone and adrenal metastasis. The patient achieved partial response after using savolitinib, maintaining a progression-free status for 12 weeks.

Currently, chemotherapy, targeted therapy, and immunotherapy are widely used for advanced gastric cancer. Recently, PD-L1 and PD-1 inhibitors have been the primary immunotherapies for lung cancer, malignant melanoma, and gastric cancer. In 2017, nivolumab was approved in Japan for patients with unresectable advanced or recurrent gastric cancer that had progressed after chemotherapy, becoming the first immunotherapy approved worldwide for gastric cancer. The results of the Check-Mate649^[4]^ study demonstrated that in the PD-L1 CPS ≥ 5, the combination of nivolumab and chemotherapy (FOLFOX or XELOX) was associated with superior OS compared with chemotherapy alone (mOS 14.4 vs 11.1), with manageable adverse effects. A growing number of studies suggest that prognosis and treatment of cancer depend not only on type, differentiation, TNM stage, and others but also on the tumor-specific signaling pathways. The HGF/c-MET pathway is essential in regulating multiple processes involved in tumorigenesis and multiple pathways associated with cancer and is a promising therapeutic target.

Met genomic alterations include METex14m, MET kinase domain mutations, MET amplification, and MET fusions. Some solid tumors rely on MET gene activity for tumor cell proliferation and survival. METex14m is present in 1% to 3% of NSCLC patients, making the receptor less susceptible to ubiquitination and degradation by the proteasome, resulting in sustained HGF/c-MET pathway activation. Thus METex14m is an independent oncogenic driver that occurs in NSCLC patients. Savolitinib is a novel c-MET inhibitor that exhibits a high degree of antitumor activity. The drug is approved in China for treating patients with NSCLC who develop METex14m after platinum-based chemotherapy or who are intolerant to platinum-based chemotherapy. In phase II clinical trials conducted in China, 70 patients with NSCLC were treated with savolitinib; the ORR (objective response rate) after a follow-up of 17.6 months was 49.2%, the PFS was 1.4 months, the DoR (duration of response) was 8.3 months, and the DCR (disease control rate) was 93.4%. Median overall survival (mOS) was 12.5 months. Adverse reactions were mainly liver function impairment and allergy.^[5]^ Surgery was performed on a patient with advanced lung adenocarcinoma diagnosed with stage IIIA (T2BN2M0) lung adenocarcinoma with METex14m after five weeks of treatment with savolitinib. The pathological response rate was 50%, and the postoperative pathological stage was PT1cN0N0.^[6]^ This suggests that preoperative treatment with savolitinib may prolong OS in patients with locally advanced operable NSCLC and provide the basis for a new direction in clinical management.

Primary MET amplification is seen in various solid tumors, most commonly in NSCLC (<1%–5%), gastric cancer (<1%–10%), type I PRCC (papillary renal cell carcinoma) (13%), type II PRCC (3%), colorectal cancer (2%–4%), and with a lower incidence in esophageal and hepatocellular carcinoma.^[7]^ In addition to lung cancer, savolitinib is used to treat PRCC and gastric cancer. A phase IB study that evaluated the safety and efficacy of savolitinib in advanced gastric cancer and NSCLC patients with c-MET abnormalities found that the ORR was 9.4 months, disease control rate (DCR) was 39.1% in all patients, and PR was expressed in all gastric cancer patients with MET amplification.^[8]^ Further analysis of the relationship between the curative effect and the copy number of the c-MET gene in gastric cancer showed that all patients with PR had a high MET gene copy number (GCN) expression.

A clinical study (NCT03091192) comparing savolitinib with sunitinib in MET-driven PRCC demonstrated a strong antitumor effect with median progression-free survival (mPFS 7.0 vs 5.6) and a lower incidence of adverse events (52% vs 74%).^[1]^

The VIKTORY is the first and largest platform umbrella study with 10 phase II trials, the first and most extensive study of multiple biomarker screening-guided targeted therapy in gastric cancer, which showed that the efficacy of savolitinib monotherapy in MET-amplified gastric cancer was significantly better than conventional therapy (6-week PFS rate of 80%).^[9]^ Furthermore, circulating tumor DNA (ctDNA) MET gene copy numbers (GCN) can predict the efficacy of savolitinib. The higher the plasma MET copy number, the better the effect of the savolitinib treatment. In advanced gastric cancer, patients with a MET copy number greater than 10 had an ORR of 50% and a PFS of 4 to 6 months when treated with savolitinib monotherapy. This indicates that savolitinib has good efficacy in gastric cancer patients with MET amplification. An ongoing correlative study (NCT04923932) also evaluates savolitinib in patients with MET-amplified adenocarcinoma of the esophagogastric junction.

Interestingly, Ye et al reported an advanced MET abnormal gastric cancer patient with bone marrow infiltration and extensive metastasis, which improved rapidly after taking savolitinib. The general condition remained stable and progression-free for more than 15 weeks.^[10]^ The patient we report here had MET-amplified advanced gastric cancer with metastasis and responded rapidly with a significant response to savolitinib treatment.

Balan et al found that both c-MET and PD-L1 could be significantly up-regulated and co-localized in renal cancer tissues and that using c-MET inhibitors could down-regulate the expression of PD-L1.^[11]^ This shows that c-MET is vital in tumor immune escape through the Ras-PI3K pathway and PD-L1 expression in renal cancer cells. Also, there are similar reports of esophageal and liver cancers.^[12,13]^ Chen et al evaluated c-met and PD-L1 protein expression in gastric cancer tissues in a correlation study. They found that PD-L1-CPS (combined positive score) and PD-L1-TPS (tumor cell positive score) in gastric cancer tissues were positively correlated with c-MET protein expression (*r* values of 0.496 and 0.317, *P* < .01).^[14]^

Gastric cancer is a disease involving multiple genes, and combination therapy is a promising trend in research.

## 4. Conclusion

This case report of a 35-year-old male with advanced gastric cancer, with bone and bilateral adrenal metastasis, MET gene amplification, and intolerance to conventional chemotherapy responded well with savolitinib treatment. He improved rapidly, with partial response, no progression at 14 weeks, and no noticeable adverse reactions.

## Acknowledgements

The authors would like to thank the patient’s family for providing the information about the patient.

The Internal Medicine-Oncology Ethics Committee of Shaanxi Provincial People’s Hospital approved this study, and the patient’s family members provided informed written consent to publish the case details and relevant pictures. The authors are also deeply indebted to all the tutors and teachers for their direct and indirect assistance and helpful advice. Special thanks to my friends who have put considerable time and effort into their comments on the draft manuscript. We thank *Medjaden* Inc. for the scientific editing of this manuscript.

## Author contributions

Xin-cheng wrote the original draft and initiated the investigations. Gai-li An revised the manuscript and advised on the methodology.

**Investigation:** GaiLi An, XinCheng He.

**Methodology:** GaiLi An.

**Supervision:** GaiLi An.

**Validation**: GaiLi An.

**Writing – original draft:** XinCheng He.

**Writing – review &amp; editing:** GaiLi An.

## References

[1] Choueiri TK Heng DYC Lee JL. Efficacy of Savolitinib vs Sunitinib in patients with MET-driven papillary renal cell carcinoma: the SAVOIR phase 3 randomized clinical trial. JAMA Oncol. 2020;6:1247–55. 32469384 10.1001/jamaoncol.2020.2218 PMC7260692

[2] Markham A. Savolitinib: first approval. Drugs. 2021;81:1665–70. 34455538 10.1007/s40265-021-01584-0

[3] Wen Ming X Ying W. The significance of c-MET and IGF in the development of gastric cancer and their influence on prognosis. Chin J Lab Diag. 2021;25:1319–21.

[4] Janjigian YY Shitara K Moehler M. First-line nivolumab plus chemotherapy versus chemotherapy alone for advanced gastric, gastro-oesophageal junction, and oesophageal adenocarcinoma (CheckMate 649): a randomised, open-label, phase 3 trial. Lancet. 2021;398:27–40. 34102137 10.1016/S0140-6736(21)00797-2 PMC8436782

[5] Lu S Fang J Li X. Once-daily savolitinib in Chinese patients with pulmonary sarcomatoid carcinomas and other non-small-cell lung cancers harbouring MET exon 14 skipping alterations: a multicentre, single-arm, open-label, phase 2 study. Lancet Respir Med. 2021;9:1154–64. 34166627 10.1016/S2213-2600(21)00084-9

[6] Fu M Feng CM Xia DQ. Neoadjuvant Savolitinib targeted therapy stage IIIA-N2 primary lung adenocarcinoma harboring MET Exon 14 skipping mutation: A case report. Front Oncol. 2022;12:954886. 36052259 10.3389/fonc.2022.954886 PMC9424904

[7] Guo R Luo J Chang J. MET-dependent solid tumours - molecular diagnosis and targeted therapy. Nat Rev Clin Oncol. 2020;17:569–87. 32514147 10.1038/s41571-020-0377-z PMC7478851

[8] Wang Y Liu T Chen G. Phase Ia/Ib study of the selective MET inhibitor, Savolitinib, in patients with advanced solid tumors: safety, efficacy, and biomarkers. Oncologist. 2022;27:342–e383. 35445725 10.1093/oncolo/oyab066 PMC9074963

[9] Lee J Kim ST Kim K. Tumor genomic profiling guides patients with metastatic gastric cancer to targeted treatment: the VIKTORY umbrella trial. Cancer Discov. 2019;9:1388–405. 31315834 10.1158/2159-8290.CD-19-0442

[10] Ye W He L Su L. Case report: prompt response to Savolitinib in a case of advanced gastric cancer with bone marrow invasion and MET abnormalities. Front Oncol. 2022;12:868654. 35444940 10.3389/fonc.2022.868654 PMC9013970

[11] Balan M Mier y Teran E Waaga-Gasser AM. Novel roles of c-Met in the survival of renal cancer cells through the regulation of HO-1 and PD-L1 expression. J Biol Chem. 2015;290:8110–20. 25645920 10.1074/jbc.M114.612689 PMC4375468

[12] Chun HW Hong R. Significance of PD-L1 clones and C-MET expression in hepatocellular carcinoma. Oncol Lett. 2019;17:5487–98. 31186768 10.3892/ol.2019.10222 PMC6507339

[13] Kim R Keam B Kwon D. Programmed death ligand-1 expression and its prognostic role in esophageal squamous cell carcinoma. World J Gastroenterol. 2016;22:8389–97. 27729745 10.3748/wjg.v22.i37.8389 PMC5055869

[14] Hu C Jian Ping H Chang Yin F. Correlation between PD-L1 and c-Met in gastric cancer and its clinical significance. Chin J Diag Pathol. 2022;29:395–400.

